# Supplementary material for: Does the regulatory quality matter in the relationship between climate finance and inclusive growth in Africa?
Source: PLoS One. 2026 Feb 27;21(2):e0332650. doi: 10.1371/journal.pone.0332650 (PMC12948123; doi:10.1371/journal.pone.0332650)
Supplement: Attachment — Submitted filename: Plos one climate Response to R1 Reviewer.docx [file pone.0332650.s002.docx]

**Responses to concerns from Reviewer #1:**

1. The identified research gap lacks clarity and needs improvement; the introduction repeatedly asserts that previous studies have not examined regulatory quality in the context of the climate-finance–inclusive-growth nexus. However, the literature review does not offer definitive systematic evidence demonstrating that regulatory quality has been entirely overlooked in governance-focused climate-finance research.

**Responses**

Dear Reviewer #1, I would like to appreciate your suggestions and comments that improved the revised manuscript. Based on your comments, the clarity of the research gap is enhanced. A lot of improvements were made. The literature review section is improved by adding the research gap and the literature gap. Bade don your good comments, the revised manuscript has provided, the definitive evidence that the issue of regulatory quality is overlooked. ( see section 1 page 2 and 3 of the revised manuscript, Section 2 Page 11 of the revised manuscript).

2. The study presents a degree of novelty; however, the emphasis on regulatory quality as a moderating variable is not fully substantiated. The theoretical rationale explaining the influence of regulatory quality on the transmission mechanism between climate finance and inclusive growth remains insufficiently articulated.

**Responses**

Dear Reviewer, I would like to appreciate you once again for your nice comments. Accepting your comments positively and strongly, the revised manuscript has fully substantiated by the studies on regulatory quality, climate finance, and economic growth. Further, your comment enabled this manuscript to deserve sufficient articulation. (See section 2.3, 7 of the revised manuscript). Thank you.

3. The contribution to the empirical literature appears to be overstated, especially given that many cited studies have already explored governance, institutional indicators, or policy quality in connection with climate finance or economic outcomes, which lessens the study's presentation of a completely new perspective. Author can improve the contribution part.

**Responses**

Dear Reviewer, based on your comments, the over-stated contribution statement has been calmed down in the revised manuscript. The existing studies on the areas of regulatory quality, climate finance, and growth have gained the appropriate recognition. I would like to ask apology on this issue. Accordingly, the author improved the contribution part in the revised manuscript (section 1, see page 3, last paragraph of the revised manuscript).

4. The relationship among climate finance, regulatory quality, and inclusive growth remains inadequately explored in the existing literature. The analysis examines these concepts in isolation rather than integrating them to illustrate the impact of regulatory quality on the equity-focused developmental results of climate finance.
   The justification for focusing on Africa as a unique empirical context is not sufficiently compelling; author need to add more justification for inclusion of Africa.

**Responses**

Dear reviewer, thank you so much for raising such an essential suggestion to enhance the revised manuscript. Based on your good comments, the revised manuscript has addressed all of your concerns on this. Accordingly, sufficient literatures were reviewed. This is done following your comments. The justification of the study is also revised in the revised manuscript. (see Section1 page 2 and 3, Section 2, pages 2.2 and 2.3, page 6 of the revised manuscript).

5. Minor:

a. Maintain consistency in words in some places, Log is in capital, and in some places it is small

**Responses**

Dear reviewer, this is done following your good comment. Proofreading concerning the Log and other related issues has been solved. The author conducted a careful proofreading of the manuscripts.

b. Author can include a robustness estimate.

**Responses**

Dear reviewer, the revised manuscript included robustness estimate in the result analysis. The model diagnoses section is included in section 4.8, page 24 of the revised manuscript. In addition, all standard errors of the model are estimated using robust standard errors.

c. In the introduction author mentioned an interactive variable abbreviation without explaining. Author can enrich the literature by adding the following recent studies for improving methodology and literature review.

**Responses**

Dear reviewer, I would like to appreciate your suggestion very timely and recent, to enhance the methodology and literature section of the revised manuscripts.

Based on your comments, two of the studies, the revised manuscript cited the following studies. (see the reference section). Thank you once again.

- Does climate policy uncertainty abate financial inclusion? An empirical analysis through the lens of institutional quality and governance. Sustainability, 17(2), 520.
- The moderating role of governance, banking regulation, and supervision on shadow economy, financial inclusion, and financial stability nexus: a case of G5 economies. Economic Change and Restructuring, 57(6), 176.

d. The author can work on the policy recommendation and future research direction. **Responses**

Dear reviewer, based on your comments, the revised manuscript has improved the policy recommendation and future research. In addition to this, the revised manuscript has incorporated the limitations of the study. (See pages 28, 29, and 30 of the revised manuscript).

**End of responses to Reviewer #1**

**Responses to concerns from Reviewer #2**

Reviewer #2: The manuscript investigates the effects of climate finance on inclusive growth in Africa and examines whether regulatory quality moderates this relationship. Using balanced panel data for 54 African countries from 2013–2023 and employing a two-step System GMM estimator, the study finds that climate finance positively influences inclusive growth in both the short and long run. Regulatory quality weakens and in fact reverses the positive effect of climate finance on inclusive growth. Several control variables (trade, capital formation, population, and external debt) exert varying short- and long-run effects. The study claims originality by incorporating a climate-finance–regulatory-quality interaction term in an inclusive growth framework. While the study is topical and contextually relevant, the following issues need to be addressed before going forward.

**Responses**

Dear reviewer, thank you so much for understanding the manuscript. Exactly, the study is what you shortly articulated. Thank you for recommending that the study be topical and contextually relevant. Your understanding of cross section of the study (54) and the time of the study was amazing and correct. The model and the finding you summarized is also right. Thank you so much!

1. The theoretical justification for why regulatory quality should moderate the climate-finance–inclusive-growth link is underdeveloped.

**Response**

Dear Reviewer #2, thank you so much for providing very imperative suggestions to improve the manuscript. Based on your comments, the revised manuscript has organized the literature review section, emphasizing the theoretical grounds of the study. In addition to this, the literature review section was well organized in a thematic way. (Sections 2.2 & 2.3 page 5 and 6 of the revised manuscript). The Literature review section is well rewritten and organized in a more scientific way.

2. The literature review is lengthy but unfocused; it mixes descriptive information with unrelated empirical findings and lacks a conceptual framework.

**Responses**

Dear reviewer, I would like to appreciate you for raising these essential questions. Based on your good comments, the literature review is now well arranged and synthesized carefully (see pages 5 and 6 of the revised manuscript). Further, Table 1 on page 10 of the revised manuscript has synthesized the literature. Further, the conceptual framework is developed in the revised manuscript. ( see page 11 of the revised manuscript). Further, the gap in the literature is well identified. (see page 11 of the revised manuscript). Thank you once again.

3. Key constructs (inclusive growth, climate finance, regulatory quality) are described, but the connection among them is weak, and no mechanism is clearly articulated.

**Responses**

Dear reviewer, I would like to appreciate you for your nice suggestions. Based on your good comments, the theoretical grounds and the connections between the constructs are well organized. (See sections 2.2 and 2.3, page 5&6 of the revised manuscript).

4. There seems to be serious issues with the data development. For example, the study uses Net Official Development Assistance received as a proxy for climate finance. This is not an accepted or valid measure unless proven with citations of other credible and relevant studies that have used it. ODA contains many unrelated components (health, education, peacekeeping, governance, social sectors). This undermines the validity of the findings and threatens internal consistency.

**Responses**

Dear reviewer, this is a very critical suggestion. Thank you for rising. Accepting your comments positively, the revised manuscript has developed the section that supports the use of climate-based ODA as the measure of climate finance. (see section 3.2, page 11 of the revised manuscript). Thank you!

5. There are also methodological and econometric issues that need to be resolved
(a) Tables report extremely large or abnormal z-values (e.g., 4.6e+12), which suggest numerical instability, scaling problems, or incorrect specification.

**Response**

Dear reviewer, based on your good comments, the revised manuscript has addressed issues related to z-values. In addition to this, the revised manuscript revisited the methodology and corrected all issues around your comments. Thank you once again (see Table 10, page 26 of the revised manuscript).

(b) There is a potential for over-instrumentation risk in the model. Though the author notes 26 instruments for 54 groups, no discussion is provided on collapsing instruments or addressing instrument proliferation, which can weaken Hansen tests in System GMM.

**Responses**

Dear reviewer, thank you so much for your golden suggestion. Based on your comment, the revised manuscript has introduced the instrumentation strategy. The issue of instrument proliferation and the collapse option are used to eliminate the problem. This shows that the model employed in the study is free from the risk of instrumentation. ( see section 4.8, page 25 of the revised manuscript).

(c) The paper incorrectly interprets IPS and LLC tests, reporting significance as “free from random walk,” which is not accurate. A clearer explanation is needed.

**Response**

Dear Reviewer, thank you once again for the comment that is basic for the analysis. Based on your good comments, in the revised manuscript, the interpretation of unit root tests is corrected accordingly (see page 19 of the revised manuscript, all is highlighted).
6. There are inconsistent variable definitions and misleading interpretations. For example, the dependent variable is “inclusive growth index (IGIN)”, yet the study keeps referring to it as “economic growth”. These are conceptually different variables.

**Responses**

Dear reviewer, thank you so much for your suggestion that improved this manuscript. Based on your comment, the revised manuscript has improved all issues of inconsistency related to the variables. As you said, the dependent variable of the study is inclusive growth (IGIN), which is measured by the inclusive growth index. Thank you once again.
7. Using logarithms on variables that contain zeros or negative values such as regulatory quality which ranges from −2.5 to +2.5 is mathematically invalid, unless adjusted.

**Reponses**

Dear reviewer, this is another nice question that improved the current study. Based on your good comments, the revised manuscript has included the paragraph that explains the transformation of the values that contain negative numbers. (See page 15 of the revised manuscript.

8. Interaction term log(CLIF*REGU) is problematic due to negative REGU values.

**Responses**

Dear reviewer, this issue is solved as the linear transformation strategy is used ( as seen on page 15 ) of the revised manuscript. Thank you so much!

9. Although the study claims originality by interacting the term of climate financing and regulatory quality (CLIF*REGU). This is common in development economics literature. The manuscript should better articulate its contribution relative to: Institutional quality literature, climate finance governance, and inclusive growth.

**Responses**

Dear reviewer, thank you so much for raising this question. As you said, the issue of climate financing is common in economic literature. However, this study emphasized the regulatory quality component of governance, which only a few of the existing literatures considered as a moderator between the climate finance and inclusive growth. This is especially relevant in Africa, where the quality of the regulation is questionable. On top of that, in Africa, there are no sufficient studies that introduced regulatory quality in Africa’s inclusive growth model. Thus, this study adds some positive drop to the existing literature on this matter.

**End of responses to Reviewer #2**

**Responses to concerns from Reviewer #3**

Reviewer #3: I commend the author for putting this manuscript together. However, a few issues abound.

**Responses**

Dear Reviewer #3, thank you for providing me with an opportunity to organize the study. Based on your good comments, the revised manuscript has restructured the literature sections.
1. The data and scope of the study was not well explained.

**Responses**

Dear reviewer, thank you so much for providing me with very essential input that improved the paper. Based on your good comments, the data of the study is well elaborated. (See page 11 of the revised manuscript. Further, the revised manuscript included a paragraph that explains the scope of the study in the introduction section of the manuscript. (see page 3, the last highlighted paragraph in yellow.

2. The literature review was scanty and for publication, the literature review approach should be at least a thematic analysis.

**Responses**

Dear Reviewer, thank you so much for raising these great suggestions. Three of your work was cited in this manuscript. I would like to appreciate you for your wonderful contribution to the advancement of science in climate finance. Frankly speaking, I am proud to be with you in the world of research. Your three works cited in the revised manuscript are:

Doku, I., Richardson, T. E., & Essah, N. K. (2022). Bilateral climate finance and food security in developing countries: A look at German donations to Sub‐Saharan Africa. *Food and Energy Security*, *11*(3). <https://doi.org/10.1002/fes3.412>

Doku, I., & Phiri, A. (2024). Climate finance and women-hunger alleviation in the global south: Is the Sub-Saharan Africa case any different? *PloS One*, *19*(2), e0290274. <https://doi.org/10.1371/journal.pone.0290274>

Doku, I., Ncwadi, R., & Phiri, A. (2021). Determinants of climate finance: Analysis of recipient characteristics in Sub-Sahara Africa. *Cogent Economics & Finance*, *9*(1). <https://doi.org/10.1080/23322039.2021.1964212>

3. The study is similar to Doku, I. (2022). Are developing countries using climate funds for poverty alleviation? Evidence from Sub-Saharan Africa. The European Journal of Development Research, 34(6), 3026-3049. I will suggest that, the author should introduce something new.

**Responses**

Dear reviewer, thank you for raising this question and suggestion. It is an imperative question. I would like to also appreciate your high-quality paper on this matter. For clarity, I would like to elaborate on the basic differences between Doku, I. (2022), and this current study.

Table 1: The distinction between Doku (2022) and the current study

| **Aspects of difference** | **Doku (2022)** | **This study** |
| --- | --- | --- |
| Data-wise difference | 2006–2017 | 2013-2023 |
| Country coverage | 44 Sub-Saharan Africa | 54 African countries |
| Dependent variable | GDP per capita and social inequality. | Inclusive growth |
| Mediating variables | regulatory quality & control of corruption | regulatory quality |

From, this Table 1, it is possible to understand that the current study has differences in time, data, and its nature, coverage of the cross-section, etc. While appreciating the high quality work of Doku (2022) in this regard, I would like to kindly rebuts that the current study is different for the existing studies in many ways of which points in the above table are some of them.

**End of responses to Reviewer #3**

**I would like to appreciate all the reviewers.**
