# Supplementary material for: Does the regulatory quality matter in the relationship between climate finance and inclusive growth in Africa?
Source: PLoS One. 2026 Feb 27;21(2):e0332650. doi: 10.1371/journal.pone.0332650 (PMC12948123; doi:10.1371/journal.pone.0332650)
Supplement: Attachment — Submitted filename: Responses to Reveiwers R2.docx [file pone.0332650.s003.docx]

**Responses to concerns from Reviewers**

**Reviewer #1:** I am satisfied with the revised version and recommend acceptance in its current form. Best Wishes.

**Response**

Dear Reviewer #1, I truly appreciate your positive assessment of the revised version and thank you for recommending the manuscript for acceptance in its current form. I would also like to express my sincere gratitude to the reviewer for the valuable insights and constructive feedback provided during the review process. Your comments greatly contributed to improving the clarity and overall quality of the manuscript.

**Reviewer #2:** Thank you for considering my comments in improving the quality of your manuscript. Continue improving on the manuscript until it fully meets the journal standard.

**Response**

Dear Reviewer #2, thank you for acknowledging our efforts in addressing your comments and recommending my work for acceptance of my work in the venerated Plos One journal. I also sincerely appreciate your thoughtful comments and constructive feedback, which have greatly contributed to improving the overall quality of this manuscript. Your insights have been invaluable throughout the revision process, and I am grateful for the time and expertise you dedicated to reviewing my work. Finally, let me promise you that I will remain committed to continuing improvements to ensure that the manuscript fully meets the standards of the journal.

**End of Responses to Reviewer #1 and #2**

Reviewer #3: The authors did their best to address most of the issues rose; however a few things needed to be relooked at.
1. In the abstract, the authors mentioned that a 1 percentage change will lead to an increase in inclusive growth by 0.3607 percent. Will that be the case if climate finance reduces? Since they kept using change.

**Responses**

Dear Reviewer #3, thank you for rising very interesting suggestion regarding the interpretation of the coefficients in the introduction section. Based on your good comments, the revised manuscript has captured your concerns and the sentences in the abstract section are correctly interpreted. This is highlighted it in yellow. The opposite might not be true based on the system GMM estimation. Thank you once again (see line 7 of the abstract in the revised manuscript).

2. The work needs serious editorial attention; I will suggest they give the work to a professional editor to work on. For instance, under literature review, they kept writing sentences like; "inclusive growth is that is, ". On page 9, they wrote "Table 1 shows the effect of climate finance on inclusive growth is the major point of debate among although the debate is hot in the current literature". I think part of the sentences was cut away in generating the pdf. version too.

**Responses**

Dear Reviewer #3, thanks you so much, for your suggestion. Based on your good comments, the revised manuscript is carefully edited and proof read has happened to improve readability and clarity (see section 2 pages 3 and 4 of the revised manuscript, highlighted yellow for instance). Thank you.
3. The GMM results didn't mention what the values in bracket represent, but I presume they are standard errors. Reviewers

**Responses**

Dear reviewer #3, thanks you so much for your very professional insight to make the manuscript high quality paper. Based on your good comments, values in bracket represents are explained as standard errors just overlapping your presumption. As a scientific writer, I would like to apologise for such silly mistakes (See Table 9, page 22 of the revised manuscript).

**End of Responses to Reviewer #3**

**I would like to appreciate all anonymous reviewers and the editor.**
